# Coûts médicaux directs de traitement du cancer du sein à l’Institut Joliot Curie de l’Hôpital Aristide Le Dantec de Dakar, Sénégal

**DOI:** 10.11604/pamj.2022.42.266.32967

**Published:** 2022-08-10

**Authors:** Mory Diallo, Dieynaba Fall, Ibrahima Mballo, Cheikh Ibrahima Niang, Mohammed Ezzet Charfi

**Affiliations:** 1 Institut Joliot Curie, Hôpital Aristide Le Dantec, Dakar, Sénégal

**Keywords:** Coûts médicaux directs, cancer du sein, traitement cancer du sein, Institut Joliot Curie, Sénégal, Direct medical costs, breast cancer, breast cancer treatment, Joliot Curie Institute, Senegal

## Abstract

**Introduction:**

en 2020, l´incidence du cancer du sein s´élevait à 2 261 419 cas dans le monde; 186 598 cas en Afrique et 1 817 cas au Sénégal. Toutefois, les coûts directs médicaux du traitement des cancers en général ne sont pas connus au Sénégal. Pour une meilleure allocation des ressources, il est important d´estimer leurs coûts. L´objectif de cet article est d´analyser les coûts médicaux directs de traitement du cancer du sein à l´Institut Joliot Curie de Dakar.

**Méthodes:**

c´est une étude rétrospective menée sur des patientes diagnostiquées d´un cancer du sein entre janvier et décembre 2017 à l´Institut Joliot Curie. Une enquête par questionnaire et des entretiens semi-structurés ont été faits auprès des personnes malades et de leurs proches pour reconstruire le coût direct médical.

**Résultats:**

le coût moyen direct médical estimé du traitement de cancer du sein à l´Institut Joliot Curie est de $3 713.45 avec un minimum de $1 495.15 et un maximum de $10 662.97 sur une durée moyenne de 31 mois. Ce coût est constitué de 29% de la chimiothérapie; de 15% du diagnostic et de 15% de la chirurgie. Les coûts de la radiothérapie et les médicaments sur ordonnance représentent 13% pour chaque acte. Ce coût médical est lié d´une part au niveau d´étude (p=0,05) et au stade de la maladie (p=0,03).

**Conclusion:**

le coût médical direct du traitement du cancer du sein est très élevé au Sénégal. Le coût médical direct du traitement maximum est de $10 662.97 et le minimum est à $1 495.15, soit un coût moyen de $3 713.45.

## Abstract (en)

**Introduction:**

in 2020, the incidence of breast cancer was 2261419 cases worldwide, 1186598 cases in Africa and 817 cases in Senegal. However, direct medical costs of cancer treatment are not known in Senegal. For a better resource allocation, it is important to estimate costs. The purpose of this study is to analyze direct medical costs of breast cancer treatment at the Joliot Curie Institute in Dakar.

**Methods:**

we conducted a retrospective study of patients diagnosed with breast cancer between January and December 2017 at the Joliot Curie Institute. A questionnaire survey and semi-structured interviews were conducted among patients and their relatives to reconstruct direct medical costs.

**Results:**

average direct medical costs of breast cancer treatment at the Joliot Curie Institute were $33 713.45 with a minimum of $1 495.15 and a maximum of $10 662.97 over an average period of 31 months. These costs include chemotherapy (29%); diagnosis (15%) and surgery (15%). Costs of radiotherapy and prescription medicines accounted for 13% for each procedure. Medical costs were related to educational level (p=0.05) and stage of disease (p=0.03).

**Conclusion:**

direct medical costs of breast cancer treatment are very high in Senegal. Direct medical costs of maximum treatment is $10 662.97 and of minimum treatment is $495.15, reflecting an average cost of $3 713.45.

## Introduction

L´incidence des cancers dans le monde s´élève à 19 292 789 cas en 2020 selon les estimations du Centre International de Recherche sur le Cancer [1]. Ces données, révèlent que 22,80% des cas de cancer se concentrent dans le continent européen; 49,26% en Asie; 13,25% en Amérique du Nord; 7,62% en Amérique latine; 5,75% en Afrique et 1,32% en Océanie. Les cancers constituent la deuxième cause de mortalité avec près d´un décès sur six et 9 958 133 de décès rien qu´en 2020 dans le monde [1]. En 2020, l´incidence du cancer du sein s´élevait à 2 261 419 cas dans le monde avec 684 996 décès. En Afrique, elle était estimée à 186 598 cas avec 85 787 décès. La mortalité est assez élevée en Afrique, certainement à cause d´un accès limité aux soins, mais aussi à cause d´un retard de consultation. En effet, on constate que 46% des personnes diagnostiquées du cancer du sein en Afrique en 2020 ont perdu la vie [1]. Le Sénégal, pour sa part, dénombre 1 817 cas avec 951 décès sur la même période [1]. L´incidence du cancer du sein est en augmentation en Afrique subsaharienne et les efforts de diagnostic précoce n´ont pas été très satisfaisants car le public connaît mal la maladie [2]. Un grand pourcentage des cas de cancer du sein sont diagnostiqués tardivement et principalement en milieu rural [2]. En Afrique subsaharienne, les défis de détermination précise des cas de cancer et de dénombrement de la population à risque rendent difficile l´obtention des données qui reflètent la réalité. La non disponibilité d’incidence réelle des cancers ne faciliterait pas une détermination du coût de la prise en charge de ces maladies. Une compréhension des éléments de coût des maladies courantes est une étape nécessaire pour garantir une utilisation optimale des ressources de santé limitées [3]. Dans la plupart des pays d´Afrique subsaharienne, le traitement du cancer n´est pas couvert par les programmes nationaux d´assurance maladie, ce qui oblige les patients à supporter le coût du dépistage et du traitement de cette maladie [2]. Le cancer touche toutes les couches de la société (jeunes et vieux, riches et pauvres, hommes, femmes et enfants). Cette pathologie, associée à des traitements lourds, provoquant la souffrance et parfois le décès, entraîne des pertes d´utilité pour les personnes malades et leurs proches, et donc une perte d´utilité sociale. Ainsi, une personne malade peut être contrainte de cesser son activité temporairement ou définitivement, ce qui a une incidence sur la production du pays en fonction de la productivité de la personne et de l´état du marché du travail.

Par ailleurs, l´ensemble des soins prodigués à des personnes ayant un cancer est très important, du fait, de la lourdeur du traitement (chirurgie, radiothérapie, chimiothérapie, bilans…), se pose alors de façon récurrente la question de savoir combien cela coûte en moyenne à la personne malade ou à ses proches à la fin du traitement? Ainsi, les soins pour les 15 types de cancer les plus répandus aux États-Unis ont coûté environ 156,2 milliards de dollars en 2018. L´équipe a également découvert que les médicaments représentaient la dépense la plus importante et que les dépenses liées aux médicaments pour les cancers du sein, du poumon, des lymphomes et colorectaux étaient les plus coûteuses. Le cancer du sein était également le type de cancer le plus cher, avec un coût total de 3,4 milliards de dollars, suivi du cancer du poumon et du cancer colorectal, qui ont tous deux été estimés à environ 1,1 milliard de dollars [4]. De même, les coûts nationaux des soins contre le cancer ont été estimés à 190,2 milliards de dollars en 2015 et à 208,9 milliards de dollars en 2020, une augmentation de 10% qui n´est due qu´au vieillissement et à la croissance de la population américaine. Ces estimations de coûts comprennent les coûts attribuables au cancer pour les services médicaux et les médicaments d´ordonnance par voie orale. Les coûts des services médicaux nationaux étaient les plus élevés pour les cancers féminins du sein. Les coûts moyens par patient étaient les plus élevés au cours de la phase de cancer de la dernière année de vie, suivie des phases initiale et continue (services médicaux: $109 727; $43 516 et $5 518 respectivement) [5]. En Afrique du Sud, le Centre d´Oncologie de Sandton s´est plaint que, bien que le coût des médicaments anticancéreux soit élevé, ils ne représentent souvent qu´une fraction du coût total. Les coûts tels que la chirurgie, les soins hospitaliers, les consultations et les examens de diagnostic et de stadification répétitifs ont fait grimper les dépenses de façon exponentielle [6]. Cependant, à notre connaissance, aucune étude n´a encore tenté d´estimer le coût des soins contre le cancer au Sénégal. Etant donné que les coûts du traitement des cancers ne sont pas connus, il serait important d´estimer et d´identifier les mécanismes de partages de ces coûts pour un accès équitable aux soins des personnes malades. En outre, cette étude est faite dans un contexte où le gouvernement a pris l´option de rendre gratuite la chimiothérapie pour les cancers du sein et celui de col de l´utérus dans les établissements publics de santé (mesure qui est entrée en vigueur le 1^er^ octobre 2019). Se pose alors la question de savoir combien s´élève en moyen le coût médical direct de traitement d´un cancer du sein au Sénégal? Par ailleurs, les coûts directs correspondent à la valeur des ressources directement consommées pour le traitement de la personne malade (le traitement du cancer du sein). On distingue deux catégories de coûts directs: les coûts directs médicaux et les coûts directs non médicaux.

Les coûts directs médicaux sont liés à l´utilisation de ressources du système de soins, ils concernent les soins délivrés par les professionnels dans des cabinets, des établissements de santé ou des officines pharmaceutiques. Les coûts directs non médicaux sont souvent à la charge du patient et concernent les frais du transport vers l´établissement de soins, ainsi que ceux liés à l´adaptation du domicile à la maladie, à l´aide domestique, à l´entretien à la restauration et à l´hébergement. Ainsi, l´objectif de cet article est d´analyser les coûts médicaux directs du traitement du cancer du sein diagnostiqué à l´Institut Joliot Curie de l´Hôpital Aristide Le Dantec de Dakar. Devant la multiplicité des structures de santé, publics et privés ayant potentiellement une activité liée à la cancérologie, il a été décidé de ne faire participer que l´Institut Joliot Curie qui est la référence pour la prise en charge des cancers au Sénégal. L´institut Joliot Curie est un institut public qui a une triple vocation à savoir les soins: l´institut est au sommet de la pyramide sanitaire dans la prise en charge des cancers en général. Il est pratiqué en moyenne 3500 consultations par an dont la majeure partie relève de pathologies tumorales ayant motivé leur évacuation à partir des structures périphériques, des services hospitaliers, des autres hôpitaux régionaux et des pays limitrophes; l´enseignement: il relève de ses attributions universitaires sur la formation des médecins en spécialisation en cancérologie et d´étudiants en médecine; la recherche: elle est motivée par l´amélioration de la prise en charge et la prévention des cancers au Sénégal et dans la sous-région [7]. En plus, une meilleure connaissance du coût de traitement de ces cancers au niveau de l´Institut Joliot Curie de l´Hôpital Aristide Le Dantec de Dakar pourrait représenter une donnée importante dans l´amélioration de politique de santé publique.

## Méthodes

L´étude est centrée au niveau des personnes atteintes d´un cancer du sein, il s´agit de savoir combien coûte pour la patiente en traitement la prise en charge diagnostique et thérapeutique du cancer du sein. La recherche a commencé par une phase d´exploration, qui a consisté à des visites d´observation en assistant aux consultations des médecins traitants, aux séances de chimiothérapie, de prise de rendez-vous des patientes. Cette phase a permis de mieux contextualiser l´étude et d´appréhender le terrain. Elle a également été mise à profit pour interroger des personnes ressources en oncologie. Ces entretiens avec les professionnels ont été réalisés en respectant le nombre minimal d´au moins un entretien par spécialité. Il s´agissait d´avoir la palette la plus large possible de professionnels en ne visant pas la saturation mais une complémentarité des entretiens.

**Population d´étude:** l´étude cible les patientes diagnostiquées à l´Institut Joliot Curie de l´Hôpital Aristide Le Dantec de Dakar d´un cancer du sein entre le 1^er^ janvier et le 31 décembre 2017. Les critères spécifiques d´inclusion suivants ont été définis: 1) avoir été diagnostiqué d´un cancer du sein entre Janvier et Décembre 2017 à l´Institut Joliot Curie de l´Hôpital Aristide Le Dantec de Dakar; 2) disposer d´un dossier médical de suivi avec numéro de téléphone au niveau de l´Institut Joliot Curie; 3) résider au Sénégal pendant la période d´étude; 4) avoir un état de santé physique et mental dont les proches jugent apte à participer à l´étude; 5) accepter de participer à l´étude. En tenant compte de ces critères, 50 patientes ont été retenues pour l´étude. Certaines données comme: le type histologique, le stade de la maladie, ainsi que le protocole de traitement incluses dans les dossiers médicaux des patientes, ont été systématiquement collectées. Le protocole de l´étude a été approuvé par le Comité d´examen éthique de l´Université Cheikh Anta Diop de Dakar. Une fiche d´information a été remise à toutes les participantes et un consentement a été obtenu de chacune d´elles. Un questionnaire leur a été administré afin de recueillir des données relatives aux dépenses afin d´établir une estimation du coût médical direct de la prise en charge de cancer du sein sur toute la période de traitement.

**Figure 1:**
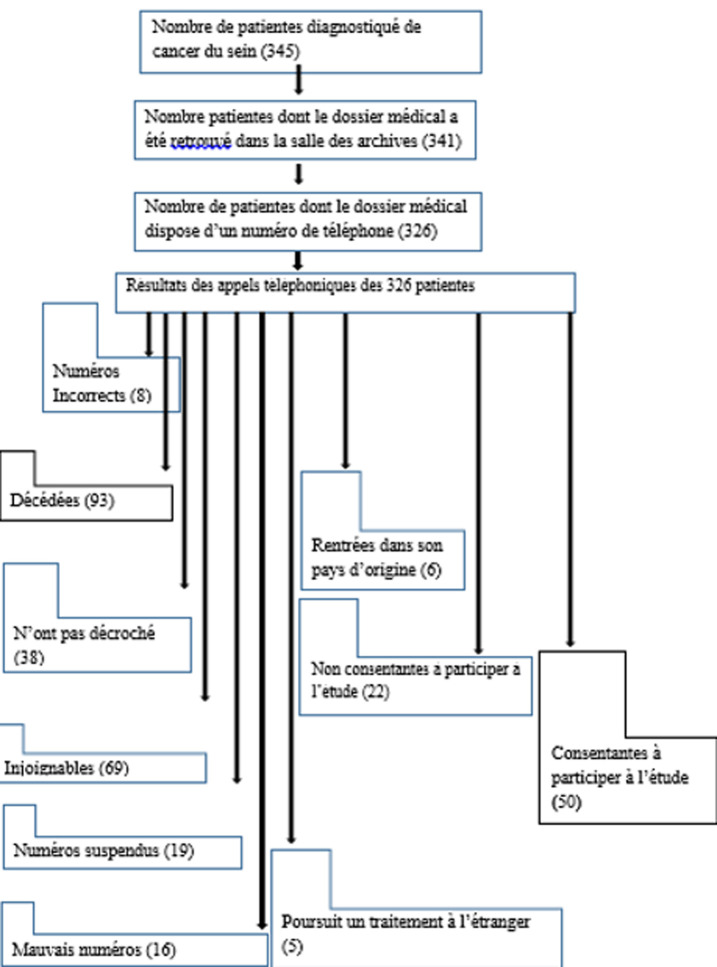
processus d’échantillonnage

**Technique d´échantillonnage:** l´échantillonnage à choix raisonné a été utilisé. Ainsi, la Figure 1 ci-dessous indique la démarche qui a été adoptée. Ainsi, ce sont au total 15,3% des patientes dont le dossier médical dispose d´un numéro de téléphone qui ont été inclus dans l´étude.

**Collecte de données:** elle a duré 9 mois. Elle a eu lieu entre le 09 décembre 2019 et le 29 Août 2020. L´étalement de la durée de collecte sur toute cette période se justifie pour deux raisons: la première est due au fait que certaines patientes résident hors de la région de Dakar. Du fait de manque de ressources pour aller régulièrement à la rencontre des patientes dans les régions, il était nécessaire de mettre à profit leurs rendez-vous à l´Institut Joliot Curie. C´est à cette occasion qu´un rendez-vous est pris au niveau de la maison d´accueil de la personne malade à Dakar pour l´administration du questionnaire. La deuxième raison est due à la pandémie du coronavirus qui a bloqué les patientes dans les régions sur toute la période de l´état d´urgence sanitaire. La majorité des patientes ont été interrogé à leur domicile afin de respecter la confidentialité. A ce niveau, le questionnaire a été administré en présence de l´accompagnant principal de chaque patiente afin de faciliter la reconstitution des dépenses effectuées dans le cadre du traitement. Exceptionnellement, certaines patientes ont été enquêtées à l´Institut Joliot Curie sur leur demande. A cet effet, des dispositions ont été prises pour l´administration du questionnaire dans le respect des critères de confidentialités. Le questionnaire a été administré sur support papier conçu avec le logiciel Sphinx plus^2^ (V5).

**Traitement et analyses:** cette phase a été réalisée avec l´utilisation des logiciels de traitement et d´analyses de données. Il s´agit entre autres d´Excel et de SPSS qui ont permis respectivement d´assurer le dépouillement, le calcul des coûts et l´analyse des variables clefs de l´étude. L´analyse descriptive a permis de ressortir les paramètres statistiques dont la moyenne, le minimum et maximum, et de procéder à des tests de corrélation. Pour faciliter la comparaison des coûts, les montants en FCFA ont été convertis en dollar américain avec un taux de change de 1 USD = 552,525 F CFA à la date du 07 avril 2021 à 00h47mn. L´équation suivante a été utilisée pour le calcul du coût médical direct: coût médical direct= coût diagnostic+coût chimiothérapie+coût radiothérapie+coût chirurgie+coût médicaments sur ordonnance+coût bilans+coût contrôle de suivi.

## Résultats

### Caractéristiques sociodémographiques des patientes de l´échantillon

**Figure 2:**
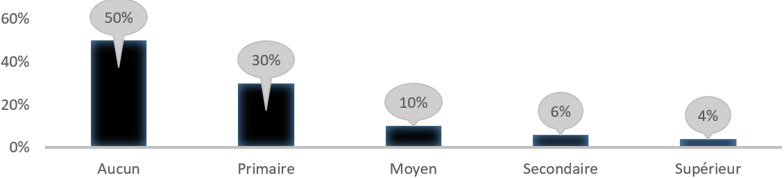
répartition des patientes selon le niveau d’instruction

**Âge et niveau d´instruction:** parmi les 50 enquêtées, 14% déclarent un âge compris entre 25 et 34 ans. Cependant 18% des patientes sont âgées de 60 ans et plus. La tranche d´âge la plus représentée est celle comprise entre 35 et 59 ans avec 68%. L´âge moyen des patientes enquêtées est de 48 ans avec un minimum de 25 ans et un maximum de 71 ans. La Tableau 1 présente la répartition des patientes selon leur classe d’âge. L´observation de la répartition selon le niveau d´instruction montre que 48% des patientes déclarent n´avoir aucun niveau d´études. De même, 30% des patientes révèle avoir un niveau d´étude primaire. Le moyen et le secondaire représentent respectivement 12% et 6% des enquêtées. Seulement 4% des patientes ont le niveau d´étude supérieur. Ainsi, la Figure 2 présente la répartition des patientes selon leur niveau d´instruction.

**Tableau 1:** répartition des patientes selon la classe d’âge

Age (classes)	(25-34 ans)	(35-59 ans)	60 ans et plus
**Effectif**	7	34	9
**Pourcentage**	14%	68%	18%

**Profession et situation matrimoniale:** pour la répartition des patientes selon la profession, les commerçantes représentent 30% de l´échantillon. Elles sont suivies par les femmes au foyer qui représentent 18%. Les ménagères et les restauratrices représentent chacune 10%. Les couturières constituent 8% de l´échantillon. D´autres professions sont aussi représentées à hauteur de 24%. Il s´agit d´enseignantes, d´agricultrices, des sage-femme, d´infirmière, d´informaticienne, d´étudiante… La répartition des patientes selon la situation matrimoniale révèle qu´elles sont majoritairement mariées avec 70% des enquêtées. Parmi ces mariées, 58% sont dans un couple monogame, 8% sont dans un couple duogame et 4% sont dans un couple polygame. Les veuves constituent 16%. Les divorcées et célibataires représentent chacune 6%. Les séparées constituent 2% des personnes enquêtées.

**Figure 3:**
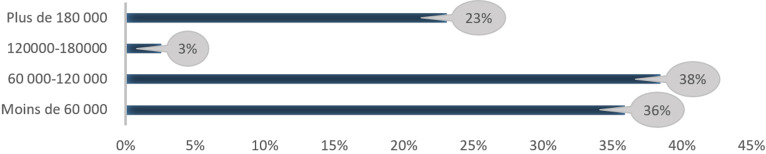
répartition des patientes selon le niveau de revenu mensuel en FCFA

**Lieu de résidence et niveau de revenu:** la répartition de l´échantillon selon le lieu de résidence révèle que la plupart des patientes résident dans la région de Dakar avec 52% des personnes enquêtées. Plus spécifiquement, ces 52% sont reparties sur les quatre département: Dakar (10%), Rufisque (10%), Guédiéwaye (12%) et Pikine (20%). Les patientes qui proviennent de la région de Thiés s´élèvent à 16%. Celles qui viennent des régions de Diourbel et de Saint-Louis représentent respectivement 10% et 6%. Les régions les moins représentées sont celles de Kolda, de Louga et de Kaolack qui constituent respectivement 4%, 4% et 2% de l´échantillon. Une faible proportion des patientes viennent d´autres régions (Ziguinchor et Sédhiou). Parmi les 50 patientes interrogées, 39 ont répondu à la question relative au niveau de revenu, soit 78% des personnes enquêtées. Parmi ces patientes, 36% révèlent disposer de moins de 60 000 F CFA en moyen par mois. Les patientes dont le niveau de revenu se situe entre 60 000 et 120 000 F CFA représentent 38%. Les personnes enquêtées qui déclarent un niveau de revenu supérieur ou égal à 180 000 F CFA représentent 23%. La tranche de revenu la moins représentée est celle comprise entre 120 000 et 180 000 F CFA avec 3% des personnes enquêtées. En outre, la Figure 3 présente les patientes selon leur tranche de revenu moyen mensuel.

**Figure 4:**
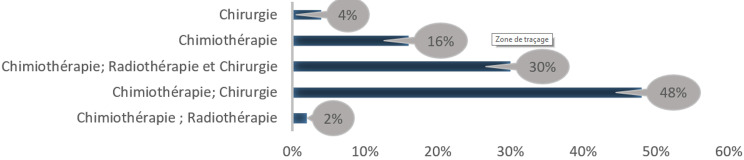
répartition des patientes selon le type de traitement

**Type de traitement, durée de traitement et stade de la maladie:** la description du traitement des patientes relève que la chimiothérapie et la chirurgie prédominent avec 48%, ensuite la chimiothérapie, la radiothérapie et la chirurgie avec 30%. Plus du quart du protocole de traitement reçu par les patientes est fait avec de la chimiothérapie soit 16%. Notons que la chimiothérapie et la radiothérapie mais aussi la chirurgie ne dépasse pas la proportion de 6% de l´échantillon. En fait, la Figure 4 fait la synthèse de la répartition des patientes selon le type de traitement. L´analyse de la durée de traitement des patientes révèle que 50% ont suivi un traitement de 31 mois et plus. Les patientes qui ont une durée de traitement comprise entre 23 et 30 mois constituent 50% de l´échantillon. La durée moyenne de traitement des patientes est de 31 mois avec un minimum de 23 mois et un maximum de 47 mois. Dans les dossiers médicaux des 50 patientes enquêtées seuls 47 disposaient des renseignements sur le stade de la maladie. La majorité des personnes enquêtées a un cancer de stade II avec 62% de l´échantillon. Les stades I et III représentent chacun 19% des enquêtées. On remarque l´absence des malades de stades IV dans l´échantillon. Le Tableau 2 fait la synthèse des patientes selon leur durée de traitement et le stade de leur maladie.

**Tableau 2:** répartition des patientes selon leur durée de traitement et le stade de leur cancer

Duré de traitement	Effectif	Pourcentage
18-23 mois	3	6%
24-30 mois	22	44%
31 mois et plus	25	50%
**Total**	**50**	**100%**
**Stade de la maladie**		
I	9	19%
II	29	62%
III	9	19%
IV	0	0%
**Total**	**47**	**100%**

### Coûts médicaux directs du traitement du cancer du sein diagnostiqué à l´Institut Joliot Curie.

**Coût du diagnostic:** l´estimation du coût du diagnostic des patientes donne les résultats suivants; on note que la tranche du coût du diagnostic comprise entre 234 000 et 286 000 FCFA est plus la représentative avec 30 % des enquêtées. Cette tranche est suivie par celle de 287 000-352 000 FCFA qui constitue 26% de l´échantillon. Le coût moyen du diagnostic du cancer du sein à l´Institut Joliot Curie est estimé à 353 895 FCFA ($640.50) avec un minimum de 158 000 FCFA ($285.96) et un maximum de 549 000 FCFA ($993.61). Dans ce coût de diagnostic, 30% est relatif au coût du scanner; 11% au coût de la mammographie; 9% au coût de l’anapathologie, l´échocardiaque, l´échographie et la biopsie représentent chacune 8%. L´électrocardiogramme et le ticket de consultation représentent chacune 2%. Ce coût de diagnostic est constitué d´autres actes médicaux à hauteur de 22%.

**Coût de la chimiothérapie:** la répartition des patientes selon le coût de leur chimiothérapie a été analysée. La tranche de coût comprise entre 645 714 et 890 000 FCFA est la plus représentée avec 31% de l´échantillon. Cette tranche est suivie par celle de 900 000 FCFA et plus qui constitue 25% des personnes enquêtées. Les patientes dont le coût de la chimiothérapie se situent entre 351 000 et 631 200 F CFA s´élève à 23%. La tranche de coût la plus faible est celle comprise entre 100 000 et 326 750 F CFA qui ne représentent que 21% des personnes interrogées. Le coût moyen de la chimiothérapie est estimé à 704 430 FCFA ($1274.92) avec un minimum de 150 000 F CFA ($271.48) et un maximum de 2 400 000 FCFA ($4343.66). L´analyse du nombre de cure de chimiothérapie donne les résultats suivants. Ainsi, 38% des personnes enquêtées ont subi entre 10 et 12 cures; 33% entre 6 et 9 cures. En outre, 21% ont bénéficié de 13 cures et plus. Cependant, seulement 8% ont eu entre 3 et 5 cures. Les personnes enquêtées qui ont bénéficié de 13 cures et plus constitue 21% de l´échantillon. Le nombre moyen de cure pour les patientes traitées par une chimiothérapie est de 10 cures avec un minimum de 4 cures et un maximum de 24 cures. Les différents protocoles thérapeutiques prescrits aux patientes ayant subi une chimiothérapie indiquent les résultats suivants: le protocole AC a été prescrit à 70% des patientes traitées par une chimiothérapie à la première séquence. Le protocole FAC a été prescrit à 15% des personnes ayant bénéficié d´une chimiothérapie. Le protocole Taxol n´a été prescrit qu´à 4% des patientes ayant bénéficié d´une chimiothérapie. Les protocoles (Taxoter, EC et Doxo-endoxan) ont été prescrits à hauteur de 11%. Pour la deuxième séquence de chimiothérapie, c´est le protocole Taxoter qui a été prescrit à 66% des patientes. Le protocole Taxol est prescrit à 24% des malades ayant reçu une deuxième séquence de chimiothérapie. Le protocole FAC n´a été prescrit qu´à hauteur de 10% pendant la deuxième séquence.

**Coût de la radiothérapie:** la majorité des patientes ont dépensé 150000 F CFA pour se faire traiter par une radiothérapie avec 94% de l´échantillon. Les personnes enquêtées qui ont dépensé 3 250 000 FCFA pour la radiothérapie constituent 6% de l´échantillon. Le coût moyen de la radiothérapie est estimé à 322 222 F CFA ($583.18) avec un minimum de 150 000 FCFA ($271.48) et un maximum de 3 250 000 FCFA ($5882.05). L´analyse du nombre de séance de radiothérapie révèle que 56% ont subi entre 21 et 25 séances. Les patientes qui ont bénéficié entre 26 et 32 séances représentent 22% des personnes enquêtées. Les patientes qui ont subi entre 33 et 36 séances constituent 17% de l´échantillon. Seulement 4% des patientes ont bénéficié de 40 séances et plus. Le nombre moyen de séance de radiothérapie est de 28 séances avec un minimum de 21 séances et un maximum de 44 séances.

**Coût de la chirurgie:** la répartition selon le coût de la chirurgie révèle que 47% des personnes enquêtées ont dépensé entre 300 000 FCFA et 369 500 FCFA. Les patientes qui ont supporté entre 250 000 FCFA et 260 000 FCFA constituent 33% de l´échantillon. Les personnes enquêtées qui ont dépensé plus de 400 000 FCFA représentent 18% de l´échantillon. Le coût moyen de la chirurgie est de 354 893 FCFA ($642.32) avec un minimum de 160 000 FCFA ($289.58) et un maximum de 1 200 000 FCFA ($2171.83). La répartition selon le coût post-opératoire révèle que 27% des patientes ont dépensé 55 100 FCFA et plus. Ce coût post-opératoire est constitué du ticket du pansement et les ordonnances relatives au pansement. Les patientes qui ont dépensé entre 42 000 FCFA et 52 500 FCFA constituent 25% de l´échantillon. Les personnes enquêtées qui ont dépensé entre 16 000 FCFA et 28 000 FCFA de coût post-opératoire représentent 25%. Seulement 23% des patientes ont supporté entre 28 600 FCFA et 41 600 FCFA des dépenses post-opératoires. Le coût moyen post-opératoire est estimé à 42 538 FCFA avec un minimum de 16 000 FCFA et un maximum de 112 500 FCFA.

**Coût des médicaments sur ordonnances:** la répartition selon le coût des médicaments sur ordonnances révèle que 32 % ont supporté 338 736 FCFA et plus. Les patientes qui ont dépensé entre 11 851 FCFA et 94 000 FCFA en médicaments sur ordonnance constituent 26% de l´échantillon. Les personnes enquêtées qui ont dépensé entre 94 798 FCFA et 187 000 FCFA représentent 24%. Seulement, 18% des patientes ont supporté entre 187 657 FCFA et 338 493 FCFA des dépenses en médicaments sur ordonnances. Le coût moyen des médicaments sur ordonnance est estimé à 305 497 FCFA ($552.91) avec un minimum de 11 851 FCFA ($21.45) et un maximum de 1 308 423 FCFA ($2368.06).

**Coût des bilans:** la répartition selon les coûts des bilans révèle que la tranche comprise entre 156 500 FCFA et 208 500 FCFA est plus représentative avec 30 %. Les patientes qui ont supporté 324 000 FCFA et plus représentent 28% de l´échantillon. Les personnes enquêtées qui ont dépensé entre 210 000 FCFA et 323 500 FCFA constituent 26% de l´échantillon. Seulement 16% des patientes ont dépensé entre 47 500 FCFA et 155 000 FCFA. Le coût moyen des bilans est estimé à 253 002 FCFA ($457.90) avec un minimum de 47 500 FCFA ($85.97) avec un maximum de 579 000 FCFA ($1047.91).

**Coût contrôle de suivi:** la répartition du coût de contrôle de suivi montre que 39% ont dépensé entre 50 000 FCFA et 105 000 FCFA. Les patientes qui ont dépensé 160 000 FCFA et plus constituent 28% de l´échantillon. Les personnes enquêtées qui ont supporté entre 5 000 FCFA et 45 000 FCFA représentent 19%. Les patientes qui ont déboursé entre 111 000 FCFA et 150 000 FCFA représentent 14% de l´échantillon. Le coût moyen de contrôle de suivi est estimé à 112 900 FCFA ($204.33) avec un minimum de 5 000 FCFA ($9.05) et un maximum de 375 000 FCFA ($678.70).

**Coût direct médical:** l´estimation du coût direct médical du cancer du sein à l´Institut Joliot Curie de l´Hôpital Aristide Le Dantec de Dakar révèle que 64% des patientes ont dépensé entre 1 294 798 et 2 343 500 FCFA. Les personnes enquêtées qui ont supporté 2 383 157 FCFA et plus constituent 26% de l´échantillon. Le coût moyen direct médical du cancer du sein diagnostiqué à l´Institut Joliot Curie est estimé à 2 051 855 FCFA ($3713.45) avec un minimum de 826 114 FCFA ($1495.15) et un maximum de 5 891 600 FCFA ($10662.97). Ce coût direct médical est lié d´une part au niveau d´étude (p=0, 05) et au stade de la maladie (p=0, 03). Le Tableau 3 suivant présente les patientes selon leur coût direct médical. Ce coût direct médical varie selon le stade de la maladie. En effet, il est de 1 993 982 FCFA pour le stade I, 2 025 490 FCFA pour le stade II et de 2 0192 014 FCFA pour le stade III. Ainsi, le coût moyen médical direct par acte est synthétisé au niveau du Tableau 4. L´analyse du coût moyen direct médical montre qu´il est constitué de 29% par la chimiothérapie. Les coûts de diagnostic et de la chirurgie représentent 15% pour chaque acte. Les coûts de la radiothérapie et les médicaments sur ordonnance représentent pour chaque acte 13%. Les bilans liés au traitement constituent 10% du coût moyen médical direct. Les dépenses liées au suivi ne représentent que 5% du coût moyen direct médical des personnes enquêtées.

**Tableau 3:** répartition des patientes selon la tranche de leur coût médical direct en FCFA

Coût direct médical	Effectif	Pourcentage
552 000-1 290 757	5	10%
1 294 798-1 674 030	16	32%
1 681 626-2 343 500	16	32%
2 383 157 et plus	13	26%
**Total**	50	100%

**Tableau 4:** synthèse du coût médical direct des patientes diagnostiquées d’un cancer du sein à l’Institut Joliot curie de l’Hôpital Aristide Le Dantec de Dakar selon l’acte medical

Actes médicaux	Minimum		Moyen		Maximum	
	F CFA	$ USD	F CFA	$ USD	F CFA	$ USD
Diagnostic	158 000	285.96	35385	640.50	549 000	993.61
Chimiothérapie	150 000	271.48	704 430	1 274.92	2 400 000	4 343.66
Radiothérapie	150 000	583.18	322 222	271.48	3 250 000	5 882.05
Chirurgie	160 000	642.31	354 893	289.58	1 200 000	2 171.83
Médicaments sur ordonnance	11 851	21.45	305 497	552.91	1 308 423	2 368.06
Bilans	47 500	85.97	253 002	457.90	579 000	1 047.91
Coût controle de suivi	5000	9.049	112 900	204.33	375 000	678.70
Coût médical direct	826 114	1 495.15	2 051 855	3 713.45	5 891 600	1 0662.97

## Discussion

**Caractéristiques sociodémographiques:** l´analyse révèle une proportion assez significative des patientes qui ont atteint l´âge de la retraite (qui est de 60 ans au Sénégal excepté de quelques professionnels) avec 14 % de l´échantillon. Cette proportion pourrait éprouver des difficultés à faire face aux dépenses liées au traitement de leur maladie. Cette catégorie des patientes pourrait donc se rendre tardivement à l´hôpital et serait susceptible de faire recours à d´autres soins moins couteux. L´âge moyen des patientes est de 48 ans. Cet âge moyen est plus élevé que ceux de Gueye *et al*. et Sarré *et al*. [8,9] qui avaient trouvé respectivement 43 et 42 ans. L´étude de Gueye *et al*. avait trouvé dans une autre série un âge médian de 29,5 ans [10]. Cette différence d´âge moyen avec celui obtenu dans cette étude serait lié au fait que Gueye *et al*. [10] avait choisi de travailler exclusivement sur les patientes âgées de moins de 35 ans. De même Gupta *et al*., Gulia *et al*., Bapna *et al*. [11-13], avaient obtenu un âge médian de 46 ans. Cet âge médian est plus proche des résultats de cette étude. Les résultats de cette étude sont en phases avec ceux de Olaogun *et al*. [14] qui avaient trouvé un âge moyen de 48,9 ans. Kakudji *et al*. pour leur part avaient obtenu un âge moyen de 56 ans, supérieur à celui de cette étude [15]. González-Robledo *et al*. démontrent que le groupe avec le plus grand nombre de cas diagnostiqués en cancer du sein était les femmes âgées entre 45 et 64 ans avec 53,5% [16]. Ces résultats sont différents avec ceux de cette étude. La plupart des patientes de cette étude (68 %) sont âgées entre 35 et 59 ans. Elles font partie de la population active et sont pour la plupart des soutiens de famille. Cette différence pourrait être liée au fait que le cancer du sein touche de plus en plus la population jeune outre, cela pourrait aussi s´expliquer par le fait que la population jeune a plus accès aux informations relatives au dépistage, diagnostic et au traitement de la maladie. En effet, cette catégorie de la population maitriserait plus les outils de communications modernes (réseaux sociaux). Un autre aspect de la littérature qui coïncide avec les résultats de cette étude est que la majorité des cas de cancer du sein détectés surviennent chez des patientes âgées de moins de 65 ans [17].

Pour le niveau d´instruction, 52% des patientes ont au moins le niveau d´étude primaire. Le niveau d´étude secondaire représente 6% de l´échantillon. Le niveau d´instruction pourrait être une variable très importante pour la sensibilisation et la prévention de cancer au sein de la famille et de la communauté. Les patientes ayant un niveau d´instruction élevé seraient plus susceptibles d´avoir accès aux bonnes informations relatives à l´importance du dépistage et de diagnostic précoce de la maladie. Elles pourraient ainsi servir de relais au niveau de leurs familles et communautés respectives. Ces résultats diffèrent de ceux de Olaogun *et al.*, Hoang *et al*. [14,18] qui avaient trouvé respectivement que 64,6% et 58,6% des patientes de leur échantillon qui avaient au moins un niveau d´étude secondaire. Dibisa *et al*. avaient obtenu une majorité des participantes (94,5%) qui étaient analphabètes contrairement aux résultats de cette étude qui ne dispose que 48% de non instruites [19]. Gómez-Rico *et al*. avaient trouvé 57,9% des femmes de niveau d´étude primaire et 23,5% qui avaient terminé la neuvième année d´étude [20]. Une étude menée en Indonésie sur la perception des risques de cancer du sein chez des lycéennes ont conclu que la plupart des participantes (69,5%) avaient un faible niveau de connaissances sur le cancer du sein. De plus, elles avaient également une perception négative (43,2%) des risques de cancer du sein. Le facteur influençant leur perception était le revenu familial (p=0,012) et les connaissances sur le cancer du sein (p=0,008) [21]. Egalement, une étude menée en Suède montre que comparativement aux femmes ayant terminé moins de 9 ans d´études, les diplômées universitaires étaient plus susceptibles de recevoir un diagnostic in situ. Les femmes ayant fait des études supérieures étaient plus susceptibles de recevoir un diagnostic de cancer du sein in situ et avaient une survie plus élevée après un diagnostic de cancer du sein invasif. Une explication de ces résultats peut être que les femmes ayant des niveaux d´éducation plus élevés participent davantage au dépistage du cancer du sein que les femmes moins instruites. Les conclusions de cette étude sont en phase avec les résultats de notre étude qui obtenu une corrélation positive entre le coût médical direct et le niveau d´instruction [22].

Par contre, une étude menée au Ghana a révélé que les étudiantes universitaires ont tendance à estimer leur risque en fonction de leur expérience du cancer du sein. Les étudiantes qui ont déjà subi un dépistage du cancer du sein et celles qui ont l´intention de procéder à un auto-examen des seins à l´avenir sont plus susceptibles de se percevoir comme étant à risque et donc de prendre des mesures pour éviter de contracter le cancer du sein. Ce qui montre que le niveau d´instruction n´a pour ce cas précis aucune influence sur la perception du risque du cancer du sein [23]. L´analyse de la situation matrimoniale révèle que les mariées représentent 70% de l´échantillon. Ces résultats sont similaires à ceux de Bambara *et al.*, Kakudji *et al*. [24,15] qui avaient obtenu respectivement 81, 25 % et 85, 5 % des mariées. Les résultats de cette étude différent de ceux de Sama *et al*. qui n´avait obtenu que 18 % des mariées dans son échantillon [25]. Salako *et al*. avait trouvé 52% des mariées, une proportion inférieur à celle de cette étude [26]. Les résultats obtenu par Gonzaga à savoir 17 % des mariées; 25 % divorcées; 58, 3 % qui ne sont pas dans un lien de mariage différent avec les données de cette série [27]. Pour le lieu de résidence, 52% des patientes résident dans la région de Dakar. Ceci est en contradiction avec les résultats de Thabane *et al*. [28] qui avaient trouvé 62,8% des résidents en zones rurales. Toutefois, les résultats de cette étude sont en phase avec ceux de Bambara *et al.*, Salako *et al*. qui avaient eu respectivement 87,50% et 80% des patientes issues de la zone urbaine [24,26].

**Coût médical direct:** le cancer est un problème de santé publique majeur au Sénégal en raison des coûts de traitement élevés. L´étude a mis en évidence que les données publiées sur ce sujet sont plutôt limitées et proviennent principalement de pays à revenu élevé, et parmi ces derniers, les États-Unis et le Canada. Les résultats de cette étude ont le potentiel de soutenir de nouvelles recherches produisant des données très utiles pour parvenir à une allocation efficace des ressources. De même, la présente étude apporte une contribution à l´information qui peut être utilisée pour la mise en œuvre d´un programme de sensibilisation d´auto-examen des seins qui faciliterait la détection précoce du cancer. Comme l´avait déjà démontré la littérature, la plupart du temps, le coût mensuel moyen du traitement dépassait largement le revenu mensuel du ménage [29]. Le coût moyen direct médical du traitement du cancer du sein à l´Institut Joliot Curie de l´Hôpital Aristide Le Dantec de Dakar est estimé à 2 051 855 FCFA sur une période moyenne de 31 mois. Sur ce, le dollar américain avec l´année de référence 2021 a été utilisé pour faciliter la comparaison des coûts. Le coût moyen estimé de 2 051 855 FCFA équivaut à $3713.45 avec un minimum de de 826 114 FCFA ($1495.15) et un maximum de 5 891 600 FCFA ($10 662.97).

Les résultats confirment que le coût médical varie en fonction du stade de la maladie. Dans cette étude, les patientes avec une maladie au stade I ont supporté en moyen 1 993 982 FCFA ($3608.83). Pour les patientes vivant avec un cancer du sein de stade II, leur coût médical moyen est estimé à 2 025 490 FCFA ($3665.85). Les patientes avec un cancer de stade III ont un coût moyen médical direct de 2 0 192 014 FCFA ($36544.72). Les coûts de traitement sont plus élevés chez les patients dont le stade du cancer est plus avancé au moment du diagnostic. Cette information peut aider à encourager les initiatives visant à renforcer le dépistage du cancer du sein. Les coûts du traitement du cancer augmentent dans le monde entier, ce qui rend les efforts de prévention et de dépistage plus économiques [30]. Les résultats de cette étude concordent avec ceux publiés par Lancet Oncology qui ont montré que le stade de la maladie au moment du diagnostic est un facteur prédictif important des coûts de traitement [31]. Les travaux de Sun *et al*. soutiennent que les coûts de traitement du cancer du sein augmentaient généralement avec l´avancement du stade de la maladie au moment du diagnostic [32]. Il a été démontré que les patients atteints d´une maladie plus avancée reçoivent plus de traitements que les patients à un stade précoce, tels que la chimiothérapie et la thérapie ciblée. La thérapie médicamenteuse est généralement une partie coûteuse du traitement pour les patients aux stades III et IV en raison de la prescription de médicaments plus coûteux [32]. Au Vietnam, d´après Hoang Lan *et al*. cité par Yohana *et al*. [33], le coût direct médical estimé du cancer du sein par patient était de $975. Ce résultat est inférieur au coût moyen estimé de cette étude qui est de $3713.45. Cela reflèterait en partie des différences de stade au moment du diagnostic ainsi que des variations dans la disponibilité et l´accès aux traitements appropriés. Le programme de surveillance, d´épidémiologie et de résultats finaux a rapporté que les cas de cancer du sein diagnostiqués à un stade précoce (stade I/II) ont un meilleur pronostic (taux de survie à 5 ans de 85 % à 98 %) [32]. Parmi les 15 études utilisant le système de stadification FIGO, les moyens des coûts de traitement cumulés pondérés par la taille des échantillons étaient de $29724 au stade I, de $39322 au stade II, de $57827 au stade III et de $62108 au stade IV. Le manque d´accès abordable à un traitement approprié du cancer du sein contribue également aux faibles coûts de traitement. Certaines patientes n´ont pas terminé leurs traitements car elles n´avaient pas les ressources financières nécessaires. De plus, les coûts unitaires peuvent être différents selon les pays [32]. Selon une autre étude menée au Mexique, les coûts de traitement différent selon le stade du diagnostic. Ainsi, les coûts de traitement des cancers aux stades I et II est de $3368, le stade III est de $5995 et le stade IV est de $5484 [16].

Une autre étude multipays a révélé qu´en Haïti les deux tiers des femmes atteintes d´un cancer du sein devaient faire face à une catastrophe financière en raison des coûts de traitement [34]. Le traitement du cancer est considéré comme le service de santé le plus coûteux en Inde. Les ménages avec des patients atteints de cancer ont dépensé 36 à 44% de leurs dépenses annuelles totales et ils pourraient perdre environ 3% de la main-d´œuvre familiale pour consacrer du temps aux soins des patients. Une étude pakistanaise a montré que le fardeau financier des soins contre le cancer était substantiel et principalement supporté par le patient ou sa famille. La plupart du temps, le coût mensuel moyen du traitement dépassait largement le revenu mensuel du ménage [29]. En Suède, le coût annuel moyen par patient pour les soins en cancer du sein était de $13238 [35] et en France (en 2004) était de $36073 [16]. Le coût annuel moyen par patient pour le secteur privé était de $15426 contre $4757 pour le secteur public [36,37]. Une étude menée au Mexique dans une institution de sécurité sociale avec des données de 2006, estime le coût moyen par an et par patient à $6734 [38]. Pour le coût moyen de traitement par acte, les résultats suivants ont été obtenus: la chimiothérapie est estimée à $1274.92, la chirurgie à $642.31 et la radiothérapie à $583.18. Ces résultats diffèrent avec ceux de González-Robledo *et al*. qui avait trouvé respectivement les coûts moyens de traitement suivants: chirurgie à $1163, radiothérapie à $376, et chimiothérapie à $6735 [16]. Les coûts moyens de chimiothérapie et de chirurgie sont plus faibles que ceux de González-Robledo *et al*. [16]. Pour ce qui est de la radiothérapie, le coût moyen de traitement est plus élevé par rapport à celui obtenu par González-Robledo *et al*. [16]. Cette variation pourrait être due à la différence des tarifs des différents actes médicaux selon les pays. Mais aussi à la différence des options politiques selon les pays. Le Sénégal a par exemple a fait l´option de subventionner la chimiothérapie des cancers du sein et celui de col de l´utérus. Toutes les patientes ayant subi une chirurgie ont reçu la mastectomie. Toutefois, cela diffère avec les résultats de Janz *et al*. qui trouvent qu´aux États-Unis qu´une plus grande proportion de femmes atteintes de cancer du sein recevait des chirurgies conservatrices [39]. Une étude menée au Ghana souligne que la plupart des pays en développement n´estiment pas le coût de la maladie et ses implications pour la croissance économique et le développement [40]. Le coût des médicaments était le coût direct le plus important de l´étude. L´étude a révélé que le coût annuel direct par patient était de 2070 GH ¢ ($358.19). Au total, le coût moyen par patient pour la période considérée était de 6008,09 GH ¢ ($1039.62) [40].

**Limite de l´étude:** notre étude présente quelques limites. Certaines patientes disposaient des ordonnances non achetées à cause d´un manque des ressources financières ce qui fait que les dépenses relatives aux médicaments sur ordonnances sont très souvent sous-estimées. En outre, d´autres patientes n´ont pas pu terminer leur traitement dû à un manque d´argent pour faire face aux dépenses liées à certains actes médicaux prescrits (chirurgie, radiothérapie…).

## Conclusion

L´objectif de cette étude est d´analyser les coûts médicaux directs de traitement du cancer du sein diagnostiqué à l´Institut Joliot Curie de Dakar. Pour une meilleure prise en charge des cancers, les coûts médicaux directs estimés payés par les personnes malades sont importants pour la planification budgétaire des soins, d´autant plus que l´assurance universelle n´existe pas au Sénégal. Le coût moyen direct médical estimé du traitement de cancer du sein est de $3 713.45 avec un minimum de $1 495.15 et un maximum de $10 662.97 sur une durée moyenne de 31 mois. Le coût médical direct du traitement du cancer du sein est très élevé au Sénégal. Cela d´autant plus que 74% des patientes de l´échantillon affirment avoir un revenu inférieur à 180 000 FCFA ($311.42) par mois. D´ailleurs une patiente témoigne sur le fardeau financier du traitement du cancer du sein en disant ceci: *« Cette maladie a ruiné ma famille »*. Sur ce l´Etat devrait: a) renforcer la subvention des médicaments relatifs au traitement de cancer; b) subventionner les scanners, c) élargir la couverture maladie universelle aux maladies à soins couteux tels les cancers, d) construire des centres de traitement de cancer dans les autres régions du pays, e) renforcer le personnel destiné à la prise en charge des cancers.

### Etat des connaissances sur le sujet

- *Le cancer du sein est diagnostiqué à un âge de plus en plus jeune en Afrique;*
- *Le cancer du sein est diagnostiqué majoritairement à un stade très avancé en Afrique;*
- *La plupart des malades ne termine pas leur traitement à cause du manque moyen financier*.

### Contribution de notre étude à la connaissance

- *Fourni une estimation du coût médical direct de traitement du cancer du sein dans un établissement public de santé du Sénégal;*
- *Fourni des données qui pourraient etre utiles à la mise en place d´un système d´assurance pour la prise en charge des cancers*.

## Remerciements

Les auteurs tiennent à remercier le directeur de l´Institut Joliot Curie de l´Hôpital Aristide le Dantec de Dakar ainsi que tous les personnels de la structure. Les remerciements vont également à l´endroit des malades, qui malgré le poids de leur maladie ont accepté de participer à cette étude. Les auteurs expriment aussi leurs profondes reconnaissances aux accompagnants et proches des personnes malades pour l´accueil, la collaboration pendant la phase de collecte et de traitement de données.
